# Comparison of treprostinil and oral sildenafil for the treatment of persistent pulmonary hypertension of the newborn: a retrospective cohort study

**DOI:** 10.3389/fped.2023.1270712

**Published:** 2023-11-03

**Authors:** Enhuan Wei, Xiu-hua Chen, Si-Jia Zhou

**Affiliations:** ^1^ Department of Neonatology, Affiliated Sanming First Hospital, Fujian Medical University, Sanming, China; ^2^ Department of Cardiac Surgery, Fujian Children's Hospital (Fujian Branch of Shanghai Children's Medical Center), College of Clinical Medicine for Obstetrics & Gynecology and Pediatrics, Fujian Medical University, Fuzhou, China

**Keywords:** treprostinil, sildenafil, pulmonary artery hypertension, PPHN, neonate

## Abstract

**Background:**

This study aims to evaluate the effectiveness of treprostinil and oral sildenafil in managing persistent pulmonary hypertension of newborns (PPHN).

**Methods:**

We conducted a retrospective cohort study of 42 neonates with PPHN treated with continuous intravenous treprostinil or oral sildenafil from January 2020 to October 2022 in China. Outcomes assessed included echocardiographic pulmonary artery systolic pressure (PASP), shunt direction, and arterial blood gas measures.

**Results:**

Treprostinil lowered PASP and improved oxygenation significantly better than sildenafil on days 1, 2, and 3 of treatment (*P* < 0.05). Treprostinil also corrected shunt direction faster than sildenafil (*P* < 0.05). The duration of mechanical ventilation, length of NICU stay, and overall hospital stay did not significantly differ between the two groups (*P* > 0.05).

**Conclusions:**

Treprostinil effectively lowers pulmonary artery pressure and improves oxygenation in neonates with PPHN, without being associated with severe complications. It may serve as a beneficial adjunct therapy for neonates with PPHN.

## 1. Introduction

Persistent pulmonary hypertension of the newborn (PPHN) is a complex medical condition marked by a challenging transitional circulatory disorder. This disorder results in heightened pulmonary vascular resistance (PVR) and causes extrapulmonary right-to-left (R-L) shunts, leading to hypoxemia (1). For afflicted neonates, the primary therapeutic objectives are prompt reduction of PVR and pulmonary artery pressure, maintaining systemic blood pressure, rectifying R-L shunts, and thereby enhancing oxygenation (2). Inhaled nitric oxide (iNO) is presently the lone pharmacological treatment for PPHN approved by regulatory authorities in the United States and the European Union. However, the response to iNO is incomplete or non-existent in approximately 30%–40% of PPHN patients (3, 4). Furthermore, the availability and application of iNO are severely limited in many hospitals within developing countries due to the lack of necessary infrastructure, medical gas sources, monitoring, and relevant technology. This necessitates the exploration of alternative therapeutic strategies (5). Prostacyclin is an endogenous substance produced by vascular endothelial cells that has potent vasodilatory, antiplatelet, and antiproliferative properties. Treprostinil, a stable analog of prostacyclin, is approved by the United States Food and Drug Administration for intravenous, continuous subcutaneous, or inhaled administration in adults with pulmonary hypertension. While treprostinil has also been used in level III NICUs (neonatel intensive care unit), evidence for its role in neonates with PPHN is limited. Sildenafil is a phosphodiesterase type 5 inhibitor with a different mechanism of action, but both have been used for pulmonary hypertension treatment (6, 7). To date, the use of treprostinil and its comparison to sildenafil for PPHN treatment have been inadequately studied. This study aimed to evaluate the efficacy of treprostinil for PPHN treatment by comparing medical data from PPHN patients receiving continuous intravenous treprostinil vs. oral sildenafil.

## 2. Materials and methods

### 2.1. Patients and study design

**Figure 1:**
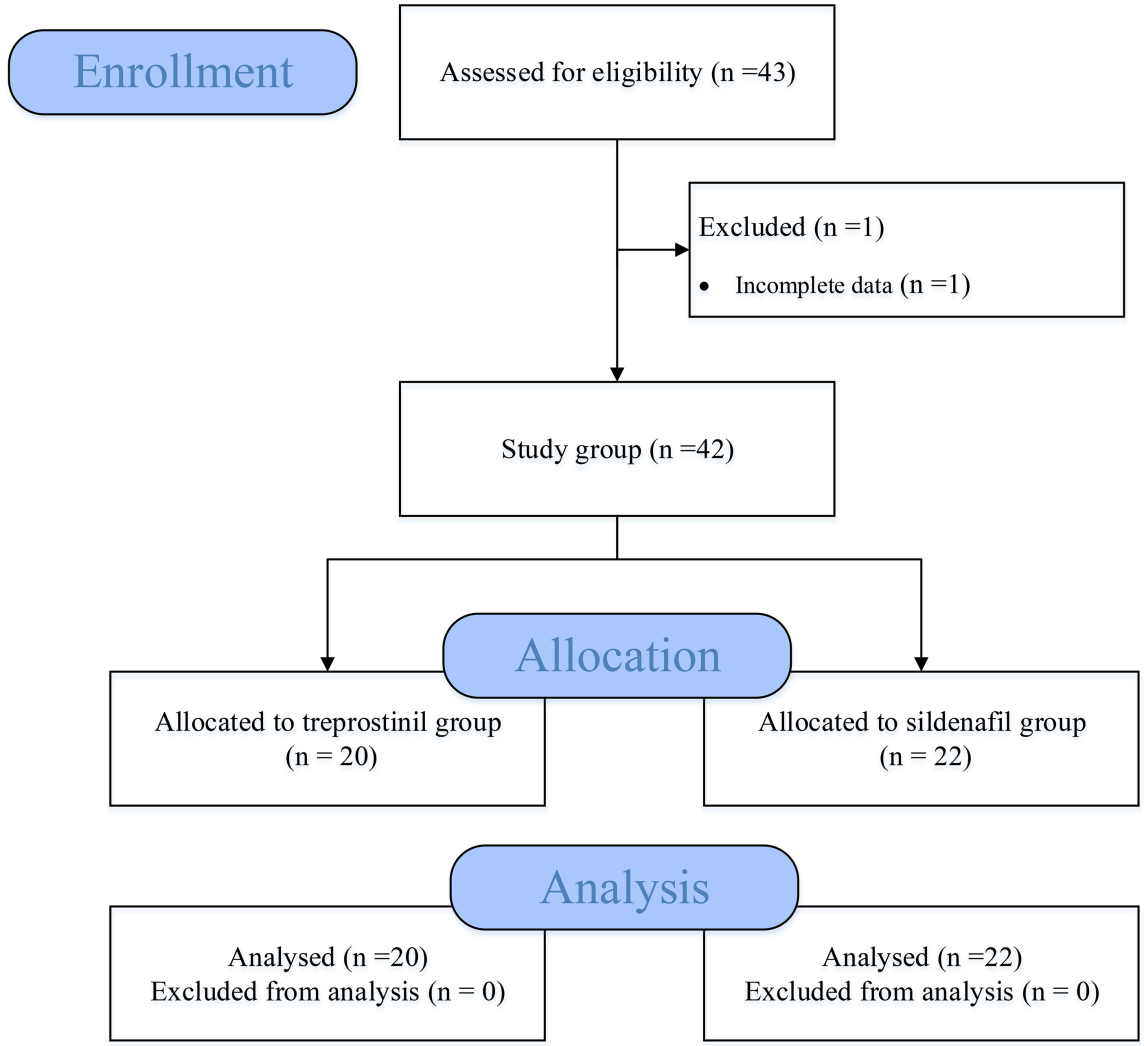
Flow diagram of the progress through the study.

We conducted a retrospective cohort study of neonates with PPHN treated in the NICU from the electronic medical record system of Fujian Children's Hospital from January 2020 to October 2022 (Figure 1). Demographic, clinical, laboratory, treatment, and outcome data were extracted from electronic medical records. Term neonates with persistent pulmonary hypertension, defined as evidence of right-to-left or bidirectional shunting on echocardiogram; pulmonary artery systolic pressure (PASP) >35 mmHg or >2/3 systolic blood pressure (sBP) pressure on echocardiogram. Exclusion criteria were: (1) Preterm infants (Defined as live-born baby born before 37 weeks of gestation); (2) Pulmonary vascular hypoplasia or dysplasia; (3) Infants with congenital heart disease or severe congenital malformations; (4) Severe birth asphyxia or any syndromes; (5) Severe PPHN requiring extracorporeal membrane oxygenation (ECMO); (6) Incomplete clinical data. This study was approved by the hospital ethics committee and informed consent was waived.

### 2.2. Echocardiographic measurements

The echocardiographic assessment of infants with persistent pulmonary hypertension of the newborn (PPHN) includes PASP, estimated primarily by the peak tricuspid regurgitant jet velocity using the modified Bernoulli equation (4V^2^), direction of blood flow across a patent ductus arteriosus (PDA) or patent foramen ovale (PFO), and position of the interventricular septum. Pulmonary hypertension severity was categorized based on the ratio of echocardiographically-derived PASP to sBP as: none/mild (PASP less than half sBP), moderate (PASP between half and equal to sBP), or severe (PASP greater than sBP) (8).

### 2.3. Therapeutic regimen

In this study, all patients were given supportive treatment for PPHN such as mechanical ventilation, oxygen inhalation, and sedation. The choice of therapeutic schedule may be based on clinician decisions at the time, the patient's household economic situation, and drug availability, among others. Patients in the treprostinil group (Group T) were given continuous intravenous pumping of treprostinil (initial dosage: 4 ng/kg/min) after the diagnosis of PPHN and the dose was gradually titrated up to a clinically effective dose, usually 20–30 ng/kg/min. During treatment, blood pressure was continuously monitored invasively, and if the blood pressure dropped below the 50th percentile for the patient's age, the treprostinil dose was decreased by 5 ng/kg/min, with dopamine given if necessary, until blood pressure stabilized. Patients in sildenafil group (Group S) were given oral sildenafil (oral sildenafil was initiated at a dosage of 0.5–2 mg/kg per dose every 8 h and titrated to a median maintenance dosage of 1 mg/kg/dose). The dosage of pulmonary vasodilator drugs may be adjusted according to the condition of the neonates by doctors during treatment. None of the patients received iNO therapy during the disease.

All patients were routinely monitored by electrocardiograph, oxygen saturation (SaO_2_), invasive arterial blood pressure, central venous pressure, and daily bedside echocardiography to assess pulmonary hypertension and appropriate frequency of artery blood gas (ABG) to assess oxygenation. ABG samples were obtained from a pre-ductal arterial line catheter in all patients. SaO_2_ measurements were derived from peripheral pulse oximetry sensors.

### 2.4. Data collection

#### 2.4.1. Basic data

The gender, gestational age, birth weight, age at diagnosis of PPHN, sBP, heart rate, inotrope use, mean airway pressure (MAP), and primary cause of PPHN were collected and recorded.

#### 2.4.2. Hemodynamic indexes

The PASP estimated and the shunt direction of oval foramen or arterial duct showed by bedside echocardiography at 3 h before (T0), 24 h (T1), 48 h (T2) and 72 h (T3) after the administration of pulmonary vasodilator drugs were recorded. The sBP at that time was also recorded, PASP/sBP was calculated, and the severity of pulmonary hypertension was graded.

#### 2.4.3. ABG indexes

The arterial partial pressure of oxygen (PaO_2_), arterial partial pressure of carbon dioxide (PaCO_2_), SaO_2_ and were recorded before (T0) and after the use of pulmonary vasodilator drugs (T1, T2, and T3). Additionally, the ventilator parameters of the fraction of inspired oxygen (FiO_2_) and MAP were also collected to calculate oxygen index (OI = FiO_2 _× MAP × 100/PaO_2_). Infants were divided into responder (infants with improved oxygenation) and non-responder (no or minimal improvement in oxygenation). The improved oxygenation was defined as a 20% increase in PaO_2_ or a 20% decrease in OI within 24 h of drug initiation (9).

#### 2.4.4. Adverse reactions and short-term outcomes

Adverse reactions that may occur during treatment, including hypotension, rash, thrombocytopenia, and impairment of liver and kidney function, should be retrieved from the electronic medical record system. The duration of mechanical ventilation, length of NICU stay, length of hospital stay, and other short-term outcomes were recorded.

### 2.5. Statistical analysis

Excel software was applied to input and collate data. SPSS Statistics 26 was used for statistical analysis. The measurement data were described by mean ± standard deviation (SD), and the normality test was performed. The t-test was used for those following the normal distribution, and the Wilcoxon rank sum test was used for those not in accordance with the normal distribution. Repeated-measures ANOVA (RM ANOVA) was used for repeated measurement data. The categorical data were described by frequency (constituent ratio) and analyzed by chi-square test or Fisher's exact test. *P* < 0.05 was considered statistically significant.

## 3. Results

### 3.1. Baseline data

A total of 43 infants were screened, of whom 1 did not meet the inclusion criteria due to incomplete data. Ultimately, 42 neonates were included in the study (20 in the treprostinil group and 22 in the sildenafil group). There were no significant differences between the two groups in gender, gestational age, birth weight, sBP, heart rate, and other baseline data (Table 1).

**Table 1:** Baseline characteristics of included neonates^a^.

Characteristics	Group T (*n* = 20)	Group S (*n* = 22)	*P* value
Gender, *n* (%)			
Male	12 (60%)	15 (68%)	0.749
Female	8 (40%)	7 (32%)	
Gestational age (weeks; mean ± SD)	39.1 ± 0.9	38.6 ± 1.0	0.063
Birth weight (kg; mean ± SD)	3.6 ± 0.9	3.3 ± 1.0	0.284
Age at diagnosis of PPHN (h; mean ± SD)	19.5 ± 8.5	21.3 ± 7.3	0.360
sBP (mmHg; mean ± SD)	71.5 ± 7.8	73.3 ± 7.0	0.445
Heart rate (beat; mean ± SD)	137 ± 12	142 ± 14	0.231
Inotrope use, *n* (%)	17 (85%)	16 (73%)	0.460
Mean airway pressure (cmH_2_O; mean ± SD)	14.5 ± 1.5	15.0 ± 1.1	0.510
Primary cause of PPHN, *n* (%)			
MAS	8 (40%)	9 (41%)	0.861
Pneumonia	5 (25%)	7 (32%)	
Idiopathic pulmonary hypertension	2 (10%)	2 (9%)	
RDS	4 (20%)	4 (18%)	
Sepsis	1 (5%)	0	

T, treprostinil; S, sildenafil; sBP, systolic blood pressure; PPHN, persistent pulmonary hypertension of the newborn; MAS, meconium aspiration syndrome; RDS, respiratory distress syndrome.

^a^ There was no significant difference in the baseline characteristics between the two groups of infants.

### 3.2. Hemodynamic indexes

The median treprostinil dose at T1 was 20 ng/kg/min (range 4–40 ng/kg/min), T2 was 25 ng/kg/min (range 10–45 ng/kg/min), and T3 was 25 ng/kg/min (range 5–40 ng/kg/min).

**Figure 2:**
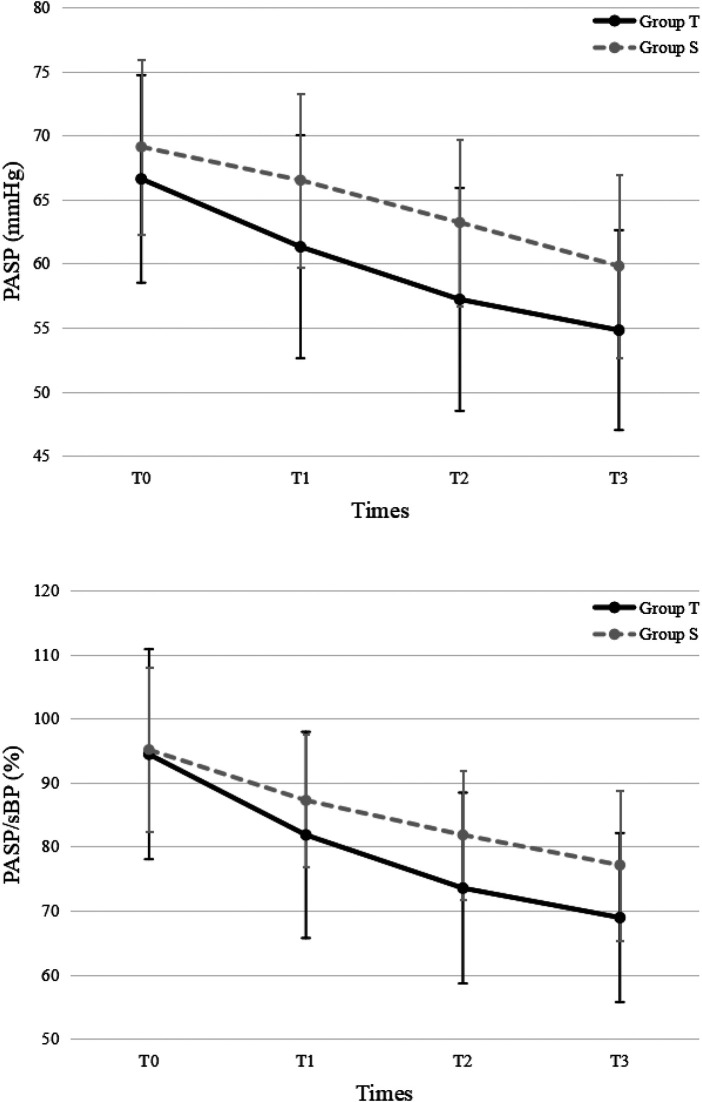
Pulmonary arterial systolic pressure. PASP, pulmonary arterial systolic pressure; sBP, systolic blood pressure; T, treprostinil; S, sildenafil.

For PASP, there were significant decreases over time in both groups (*P *< 0.001). PASP was significantly lower in the treprostinil group compared to the sildenafil group at T1, T2, and T3 (*P *< 0.05 for all) (Figure 2).

**Figure 3:**
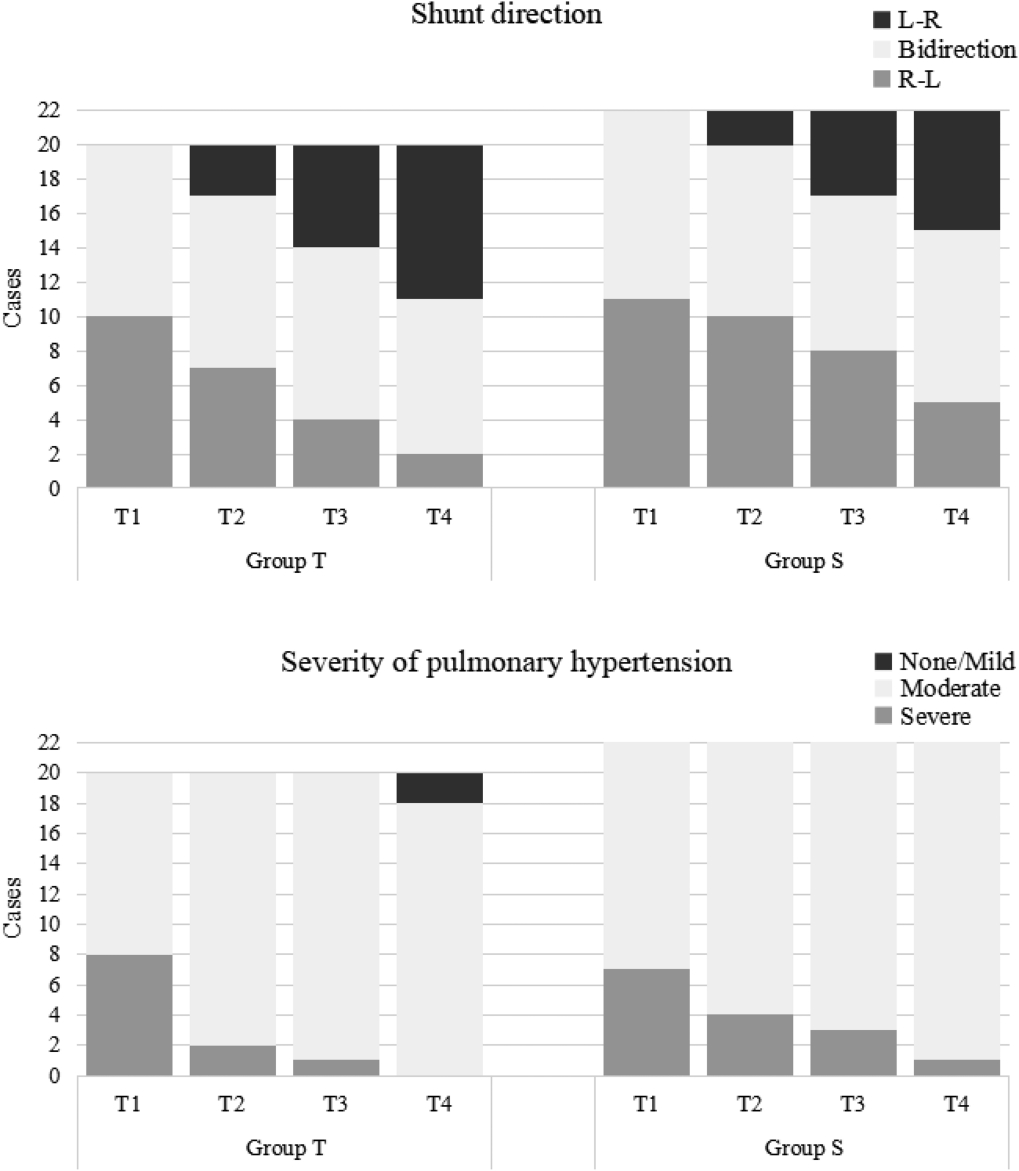
Shunt direction and severity of pulmonary hypertension; T, treprostinil; S, sildenafil.

For PASP/sBP, there were significant decreases over time in both groups (*P *< 0.001). PASP/sBP was significantly lower in the treprostinil group compared to the sildenafil group at T2 (*P* = 0.043) and T3 (*P* = 0.039) (Figure 2). Pulmonary hypertension severity was categorized based on the PASP/sBP. Significant changes occurred at T2 and T3 in the treprostinil group (*P *< 0.05) and at T3 in the sildenafil group (*P *= 0.02) compared to T0 (Figure 3).

All patients had PDA or PFO. For the shunt direction, most patients in both groups changed from right-to-left or bidirectional shunting to left-to-right shunting over time. Significant changes occurred at T2 and T3 in the treprostinil group (*P *< 0.05) and at T3 in the sildenafil group (*P *= 0.01) compared to T0 (Figure 3).

### 3.3. ABG indexes

For SaO_2_, there were significant increases over time in both groups (*P *< 0.001) but no significant difference between groups (Table 2).

**Table 2:** ABG indexes.

Variable group		T0	T1	T2	T3
SaO_2_	Group T	83.1 ± 4.6	89.8 ± 5.8	94.2 ± 4.5	96.5 ± 4.1
	Group S	80.9 ± 6.7	87.3 ± 6.1	92.4 ± 4.1	96.0 ± 2.5
	*P*	0.234	0.114	0.172	0.618
PaO_2_	Group T	57.3 ± 8.1	70.5 ± 7.9	79.1 ± 12.0	92.4 ± 19.3
	Group S	55.1 ± 7.5	65.0 ± 9.0	72.3 ± 9.2	83.1 ± 8.7
	*P*	0.360	0.041	0.046	0.049
PaCO_2_	Group T	47.2 ± 6.9	44.9 ± 10.5	44.4 ± 8.3	43.5 ± 4.1
	Group S	49.5 ± 10.4	46.6 ± 8.1	45.7 ± 4.4	45.1 ± 4.8
	*P*	0.407	0.559	0.533	0.231
OI	Group T	24.2 ± 4.7	19.9 ± 4.0	17.8 ± 2.7	15.3 ± 2.5
	Group S	25.5 ± 4.7	22.5 ± 4.0	19.8 ± 3.2	16.2 ± 3.0
	*P*	0.399	0.048	0.030	0.036

T, treprostinil; S, sildenafil; OI, oxygen index.

For PaO_2_, there were significant increases over time in both groups (*P *< 0.001). PaO_2_ was significantly higher in the treprostinil group compared to the sildenafil group at T1, T2, and T3 (*P *< 0.05 for all) (Table 2).

For PaCO_2_, there were no significant differences between or within groups (*P *> 0.05) (Table 2).

For OI, there were significant decreases over time in both groups (*P* < 0.001). OI was significantly lower in the treprostinil group compared to the sildenafil group at T1, T2, and T3 (*P *< 0.05 for all) (Table 2).

There were 12 (60%) and 9 (41%) responders in the treprostinil group and sildenafil group, respectively.

### 3.4. Adverse reactions and outcomes

Both drugs were generally well tolerated, however, one patient developed hypotension that required a dose reduction of the study drug and initiation of dopamine. Duration of ventilation, NICU, and hospital stay were similar between groups. After commencing the study drug, 10% (*n* = 2) of patients in the sildenafil group and 14% (*n* = 3) of patients in the treprostinil group subsequently required extracorporeal membrane oxygenation (ECMO) support (Table 3). All patients who underwent ECMO were successfully weaned from ECMO, but one patient death occurred in the sildenafil group after ECMO weaning due to infection (Table 1).

**Table 3:** Adverse reactions and short-term outcomes.

Variable	Group T (*n* = 20)	Group S (*n* = 22)	*P* value
Hypotension, *n* (%)	2 (10%)	1 (5%)	/
Needed new inotrope after treatment, *n* (%)	3 (15%)	2 (9%)	/
Air leak syndrome, *n* (%)	1 (10%)	0	/
Duration of mechanical ventilation (day; mean ± SD)	8 ± 5	10 ± 4	0.175
NICU stay (day; mean ± SD)	13 ± 6	14 ± 5	0.898
Length of hospital stay (day; mean ± SD)	18 ± 5	19 ± 6	0.881
ECMO support, *n* (%)	2 (10%)	3 (14%)	/
Death, *n* (%)	0	1 (5%)	/

T, treprostinil; S, sildenafil; NICU, neonatel intensive care unit; ECMO, extracorporeal membrane oxygenation.

## 4. Discussions

In treating PPHN, sildenafil is the most used phosphodiesterase inhibitor in neonates. Its efficacy is confirmed by many studies to improve oxygenation and reduce mortality (10–12). This study found treprostinil, compared to oral sildenafil, can reduce pulmonary artery pressure, and improve oxygenation of PPHN newborns, with no serious complications.

The results of this study confirmed treprostinil's effectiveness in reducing pulmonary artery pressure in PPHN neonates. Compared to baseline (T0), PASP and PASP/sBP all decreased in both treprostinil and sildenafil groups. PASP was lower in the treprostinil group at T1, T2, and T3 compared to the oral sildenafil group. PASP/sBP was lower in the treprostinil group at T2 and T3 compared to the oral sildenafil group. Although no significant difference in shunt was seen between groups, both groups showed improvement at later timepoints. Overall, intravenous treprostinil reduced pulmonary pressures faster than oral sildenafil. However, further research is needed to confirm if one medication provides superior correction of right-to-left shunting compared to the other. As Bo et al. suggested, quantitative ductal shunt assessment using velocity time integral (VTI) ratios could better risk stratify PPHN patients than qualitative shunt patterns (13). Future studies should investigate whether the incorporation of quantified ductal flow metrics can improve the identification of neonates requiring more aggressive PPHN therapy.

Sildenafil, a specific phosphodiesterase type 5 (PDE5) inhibitor, selectively relaxes pulmonary vessels, reduces pulmonary vascular resistance, improves vascular remodeling, and inhibits pulmonary hypertension. Its efficacy in neonatal persistent pulmonary hypertension has been established (7, 11). As oral sildenafil is widely used as first-line therapy for PPHN, investigating alternative administration routes could expand treatment options. Intravenous sildenafil may be advantageous in resource-limited settings where oral preparations are unreliable. Some studies suggest intravenous and oral sildenafil have equivalent efficacy in other conditions (14, 15). Comparing intravenous vs. oral sildenafil for PPHN would provide useful data on their relative effects on pressures and outcomes. If effective, intravenous sildenafil could increase PPHN treatment access in low-resource regions.

Treprostinil use in neonates is less reported, but some studies support our conclusion. Lawrence et al. conducted a retrospective cohort study and found that treprostinil was associated with improved pulmonary hypertension severity by echocardiogram and decreased BNP, with no significant side effects (16). This is similar to our results. Additionally, treprostinil has other administration routes, including oral, subcutaneous injection, and nebulization, studied for effectiveness in reducing pulmonary artery pressure (17–19). As a prostacyclin analog, treprostinil produces effects by binding prostacyclin receptors. In pulmonary vessels, it promotes cyclic adenosine monophosphate generation after binding, which opens Ca^+^-K^+^ channels, hyperpolarizes membranes, and dilates vessels (20, 21). Among prostacyclin receptors in human embryonic kidney cells, treprostinil has high affinity for PGI_2_, PGE_2_, and PGD_2_ receptors, but low for vasoconstrictor receptor (VPEP1-R). Vasodilatory and antiproliferative effects are also mediated by peroxisome proliferator-activated receptors (22).

Treprostinil's efficacy in PPHN treatment is also reflected in improved oxygenation. We used SpO_2_, PaO_2_, and OI to assess oxygenation function. Results showed improved SpO_2_, PaO_2,_ and OI in both groups with either drug. Moreover, compared to group S, although no significant SpO_2_ difference between groups, PaO_2_ was higher and OI lower in group T at T1, T2, and T3. In addition, our results show that there were 12 (60%) and 9 (41%) with improved oxygenation within 24 h in the treprostinil group and sildenafil group, respectively. Based on the above indicators, we believe treprostinil is effective for PPHN treatment. Park et al. reported 2 PPHN cases in preterm infants refractory to vasopressors, inotropes, and inhaled nitric oxide, successfully treated with treprostinil (23). This agrees that treprostinil may be a useful adjuvant therapy in PPHN neonates.

One study reported that neonates may require higher doses than conventionally used in older children (>20 ng/kg/min) (24). In our study, we started treprostinil at an initial dose of 4 ng/kg/min as a continuous intravenous infusion via a central venous line, titrating up over days to a clinically effective dose, usually 20–30 ng/kg/min. Several studies have examined the safety and tolerability of treprostinil in infants with pulmonary hypertension, although some have reported related hypotension and desaturation (25, 26). However, in our cohort, only 1 neonate developed transient hypotension after treprostinil 50 ng/kg/min, which improved after decreasing the dose to 30 ng/kg/min and adding a small dose of dopamine. We did not observe adverse events like desaturation or thrombocytopenia in this study. Therefore, the safe dosing range of treprostinil in neonates appears to vary between individuals and remains to be defined. In our study, no statistically significant difference was seen in the duration of mechanical ventilation, NICU, or hospital stay between groups. Additionally, outcomes were not statistically significant between groups. This is puzzling, as treprostinil did not appear to impact outcomes vs. oral sildenafil. Further research may be needed.

This study has several limitations. Firstly, the small retrospective sample size reduces statistical power. Secondly, the single-center design may limit generalizability of the findings to other institutions. A limitation of the echocardiographic data is that variability in equipment and echocardiographers precluded assessment of all parameters at all timepoints for each infant. Furthermore, the broad term “bidirectional shunting” represents a heterogeneous spectrum of shunting severity and directionality that could not be distinguished from the current dataset. Moving forward, large prospective multicenter randomized controlled trials are warranted to further verify these preliminary findings.

## 5. Conclusions

Treprostinil may reduce pulmonary artery pressure, improve oxygenation in neonates with PPHN, and does not appear to be associated with serious complications. It could be a useful adjuvant therapy in neonates with PPHN. However, further large sample, multicenter, prospective studies are needed to confirm the efficacy of treprostinil in this population.

## Data Availability

The raw data supporting the conclusions of this article will be made available by the authors, without undue reservation.
